# Satellite Assessment of Bio-Optical Properties of Northern Gulf of Mexico Coastal Waters Following Hurricanes Katrina and Rita

**DOI:** 10.3390/s8074135

**Published:** 2008-07-10

**Authors:** Steven E. Lohrenz, Wei-Jun Cai, Xiaogang Chen, Merritt Tuel

**Affiliations:** 1 Department of Marine Science, The University of Southern Mississippi, Stennis Space Center, MS 39529, USA; E-mails: xiaogang.chen@usm.edu; merritt.tuel@usm.edu; 2 Department of Marine Sciences, The University of Georgia, Athens, GA, 30602, USA; E-mail: wcai@uga.edu

**Keywords:** Ocean color remote sensing, hurricanes, coastal water quality, K_490, chlorophyll, MODIS Aqua, Gulf of Mexico

## Abstract

The impacts of major tropical storms events on coastal waters include sediment resuspension, intense water column mixing, and increased delivery of terrestrial materials into coastal waters. We examined satellite imagery acquired by the Moderate Resolution Imaging Spectroradiometer (MODIS) ocean color sensor aboard the Aqua spacecraft following two major hurricane events: Hurricane Katrina, which made landfall on 29 August 2005, and Hurricane Rita, which made landfall on 24 September. MODIS Aqua true color imagery revealed high turbidity levels in shelf waters immediately following the storms indicative of intense resuspension. However, imagery following the landfall of Katrina showed relatively rapid return of shelf water mass properties to pre-storm conditions. Indeed, MODIS Aqua-derived estimates of diffuse attenuation at 490 nm (K_490) and chlorophyll (chlor_a) from mid-August prior to the landfall of Hurricane Katrina were comparable to those observed in mid-September following the storm. Regions of elevated K_490 and chlor_a were evident in offshore waters and appeared to be associated with cyclonic circulation (cold-core eddies) identified on the basis of sea surface height anomaly (SSHA). Imagery acquired shortly after Hurricane Rita made landfall showed increased water column turbidity extending over a large area of the shelf off Louisiana and Texas, consistent with intense resuspension and sediment disturbance. An interannual comparison of satellite-derived estimates of K_490 for late September and early October revealed relatively lower levels in 2005, compared to the mean for the prior three years, in the vicinity of the Mississippi River birdfoot delta. In contrast, levels above the previous three year mean were observed off Texas and Louisiana 7-10 d after the passage of Rita. The lower values of K_490 near the delta could be attributed to relatively low river discharge during the preceding months of the 2005 season. The elevated levels off Texas and Louisiana were speculated to be due to the presence of fine grain sediment or dissolved materials that remained in the water column following the storm, and may also have been associated with enhanced phytoplankton biomass stimulated by the intense vertical mixing and offshore delivery of shelf water and associated nutrients. This latter view was supported by observations of high chlor_a in association with regions of cyclonic circulation.

## 1. Introduction

Satellite sensors provide a valuable tool in observing impacts of major storms on marine ecosystems. Satellite observations provide temporal continuity enabling comparisons of conditions before and after storm events and give access to areas that may otherwise not be accessible with conventional ship-based sampling techniques. Prior studies have used remote sensing observations to examine effects of hurricanes and other major storm events on coastal and oceanic water column properties. Various investigators have observed increases in satellite-derived chlorophyll concentrations in coastal (Davis and Yan, 2004; Yuan et al., 2004; Walker et al., 2005; Miller et al., 2006) and offshore waters (Babin et al., 2004; Gierach and Subrahmanyam, 2008) following the passage of hurricanes. Hu et al. (2007) reported observations by satellite of increases in sea surface reflectance and contrasting changes in sea surface temperature on either side of the storm following the passage of Hurricane Dennis in July 2005 over the west Florida shelf. They attributed the observed patterns to sediment resuspension over shallower regions of the west Florida shelf and noted that conditions recovered to pre-storm levels within about 10 d.

**Figure 1.**
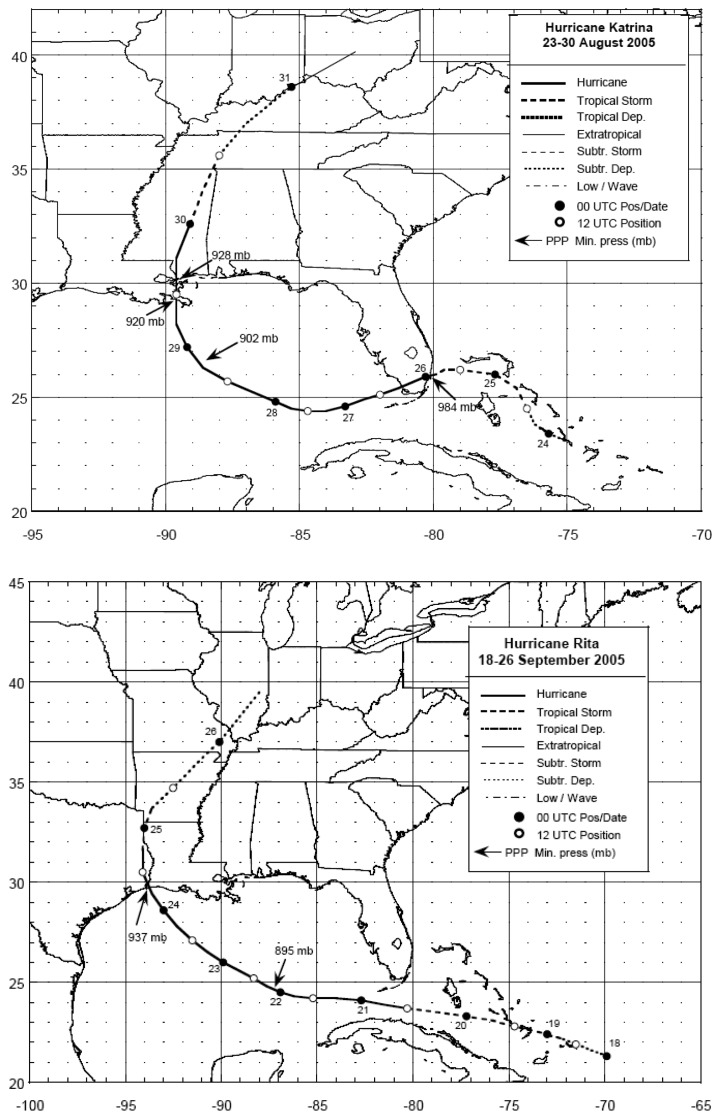
Best track positions for hurricanes Katrina (upper panel, from Knabb et al., 2005) and Rita (lower panel, from Knabb et al., 2006). Figures are reproduced by permission from NOAA.

Hurricane Katrina was a massive hurricane that first struck southern Florida as a Category 1 (on the Saffir-Simpson scale), reached Category 5 in intensity over the Gulf of Mexico and eventually made landfall as a Category 3 storm on August 29, 2005 on the northern Gulf coast near (Figure 1a; Knabb et al., 2005). Hurricane Rita attained Category 5 strength over the Gulf of Mexico and exhibited the fourth lowest central pressure for storms in the Atlantic basin. It weakened to a Category 3 storm prior to landfall on September 24, 2005 near the Texas/Louisiana border (Figure 1b; Knabb et al., 2006). Both storms had devastating societal impacts on the coastal communities in their path, but the consequences of the storms for coastal ecosystems are only beginning to be understood. Large accumulation of sediments has been reported for coastal wetlands (Turner et al., 2006). However, less is known about the impacts on coastal water quality in response to passage of the storms.

Here, we examined the satellite record prior to and immediately following the passage of hurricanes Katrina and Rita in the northern Gulf of Mexico coastal margin. Our results reinforce the view that such storms generate intense sediment resuspension and that such impacts may extend over much of the shelf and adjacent offshore waters. However, in the case of Hurricane Katrina, we found that water column conditions over the shelf within two weeks of the storm were not substantially different from that prior to the storm, and it appeared that other factors such as wind forcing and terrestrial inputs were predominant influences on water column optical properties as visible by satellite. Off Texas and Louisiana, elevated levels of diffuse attenuation appeared to persist for a longer period and were speculated to be the result of fine grain sediment or dissolved materials that remained in the water column following the storm. Increases in phytoplankton biomass stimulated by intense vertical mixing and offshore delivery of shelf water and associated nutrients may also have contributed to the higher diffuse attenuation.

## 2. Data and Methods

Satellite imagery was acquired from the Moderate Resolution Imaging Spectroradiometer (MODIS) aboard the Aqua satellite. The MODIS instrument is a hybrid cross-track scanner with a swathwidth of 2300 km and corresponding scan-angle range of ±55° providing global coverage every one to two days (Martin, 2004). The sensor has 36 spectral bands using 12 bit digitization for high radiometric sensitivity. Nine spectral bands in the visible and near infrared are used for ocean color remote sensing (bands 8-16 with center wavelengths from 412-865 nm), each with a nominal resolution of 1-km at nadir. In addition, there are a series of 500-m nadir resolution bands for observing land/cloud properties (bands 3-7) and 250-m nadir resolution bands for land/cloud boundary (bands 1 and 2). Image data were acquired from the NASA Goddard Space Flight Center Ocean Color Web (Feldman et al., 2007). Level 0 files were processed to the 250 m resolution Level 2 products using the SeaDAS data analysis system version 5.1 (Baith et al., 2001). True color images were generated from the high resolution Level 1B image files using the ‘msl1tcpcbox’ function in SeaDAS (‘l1mapgen’ in later versions). The true color images were generated from the 250-m resolution red (645 nm, band 7) band and by interpolation of the 500-m resolution blue (469 nm, 2) and green (555 nm, 6) bands. For the interannual comparisons, diffuse attenuation at 490 nm (K_490) and chlorophyll a (chlor_a) products were extracted from the Level 2 1-km resolution imagery obtained from the Ocean Color Web and mapped using SeaDAS version 5.2. The K_490 product was determined as described in O'Reilly et al. (2000) and given in Equation 1:

(1) $$K\_490=K_{w}\;(490)+A{\left[\frac{L_{w}\;(488)}{L_{w}\;(551)}\right]}^{B}\;,$$

where *L_w_*(488) and *L_w_*(551) are water leaving radiances at 488 and 551 nm, *K_w_*(490)is the diffuse attenuation for pure water (=0.016 m^-1^), *A*=0.15645, and *B*=-1.5401. The chlor_a product was determined using the OC3M chlorophyll algorithm as given in Equation 2:

(2) $$\text{chlor}\_a=10^{\left(0.283-2.753R_{3M}+1.457R_{3M}^{2}+0.659R_{3M}^{3}-1.403R_{3M}^{4}\right)}\;,$$

where *R*_3_*_M_* is the greater of either $\log_{10}(R_{551}^{443})$ or $\log_{10}(R_{551}^{488})$ and $R_{\lambda_{2}}^{\lambda_{1}}$ is abbreviated notation for the ratio of remote sensing reflectances at wavelengths *λ*_1_ and *λ*_2_. Mesoscale historical sea surface height anomaly (SSHA) data were obtained using the Colorado Center for Astrodynamics Research Gulf of Mexico Near Real-Time Altimeter Viewer (CCAR, 2008). These products are produced using information from a variety of satellite altimeters including JASON, TOPEX/POSEIDON, GEOSAT Follow-On, ERS-2, and ENVISAT. Further description of the processing procedures for the SSHA data is given in Leben et al. (2002).

## 3. Results

**Figure 2.**
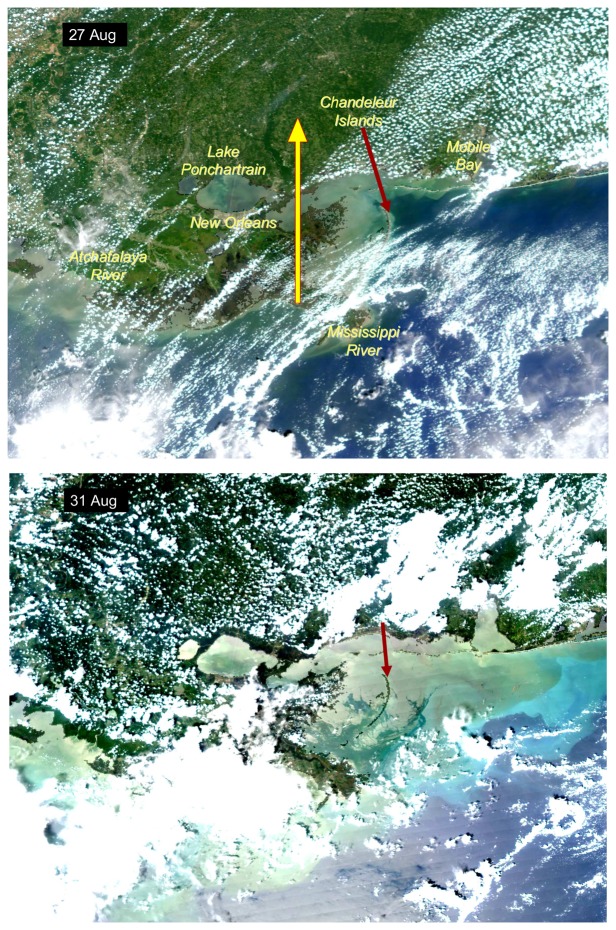
MODIS Aqua high resolution true color images of the northern Gulf coast region approximate two days prior to (top panel) and two days after (bottom panel) Hurricane Katrina made landfall. The yellow arrow in the top panel shows the approximate path of the storm as it made landfall. The red arrow indicates the location of the Chandeleur Islands as a reference. High turbidity is evident over much of the Mississippi Bight region following the storm.

**Figure 3.**
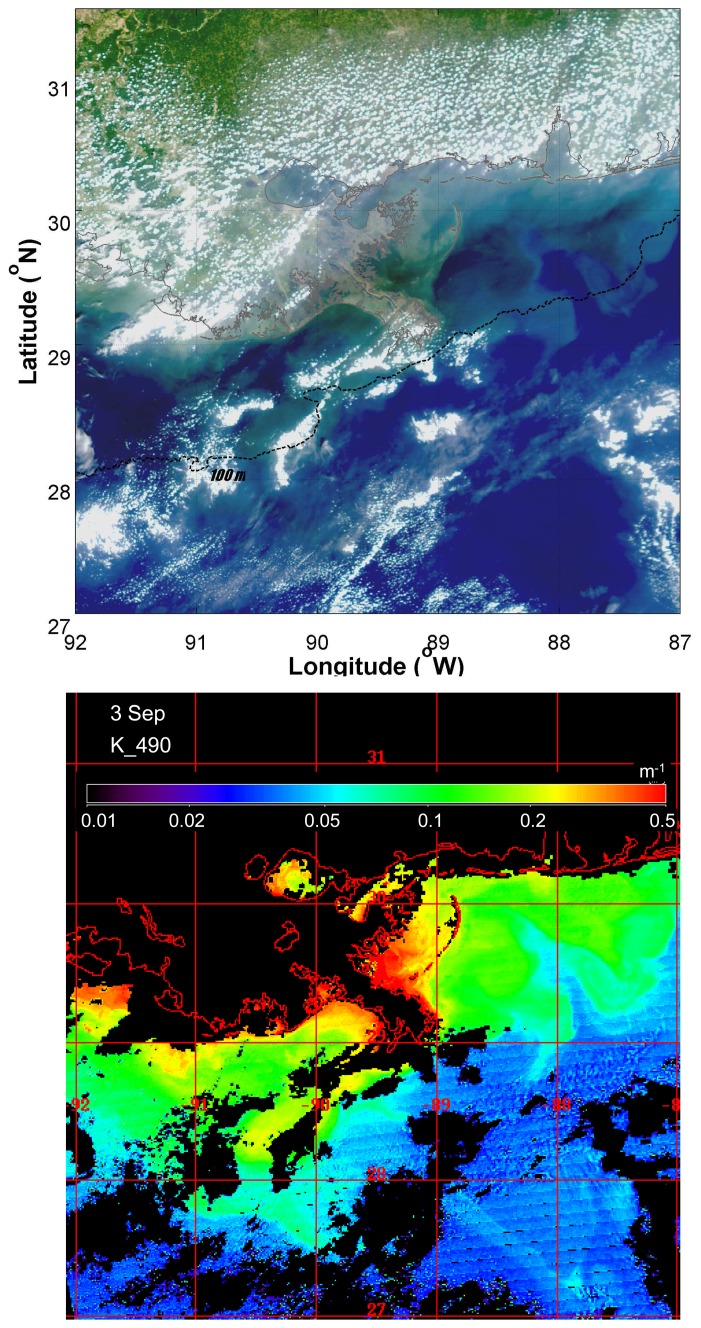
MODIS Aqua high resolution true color image of the northern Gulf coast region on September 3, 2005 (upper panel). The dashed line corresponds to the 100 m isobath, which is representative of the shelf edge. High turbidity water extending out over the shelf is evident in the lower image of diffuse attenuation at 490 nm (K_490). Units of K_490 were m^-1^. Black areas in the K_490 image are masked pixels due to clouds or land.

**Figure 4.**
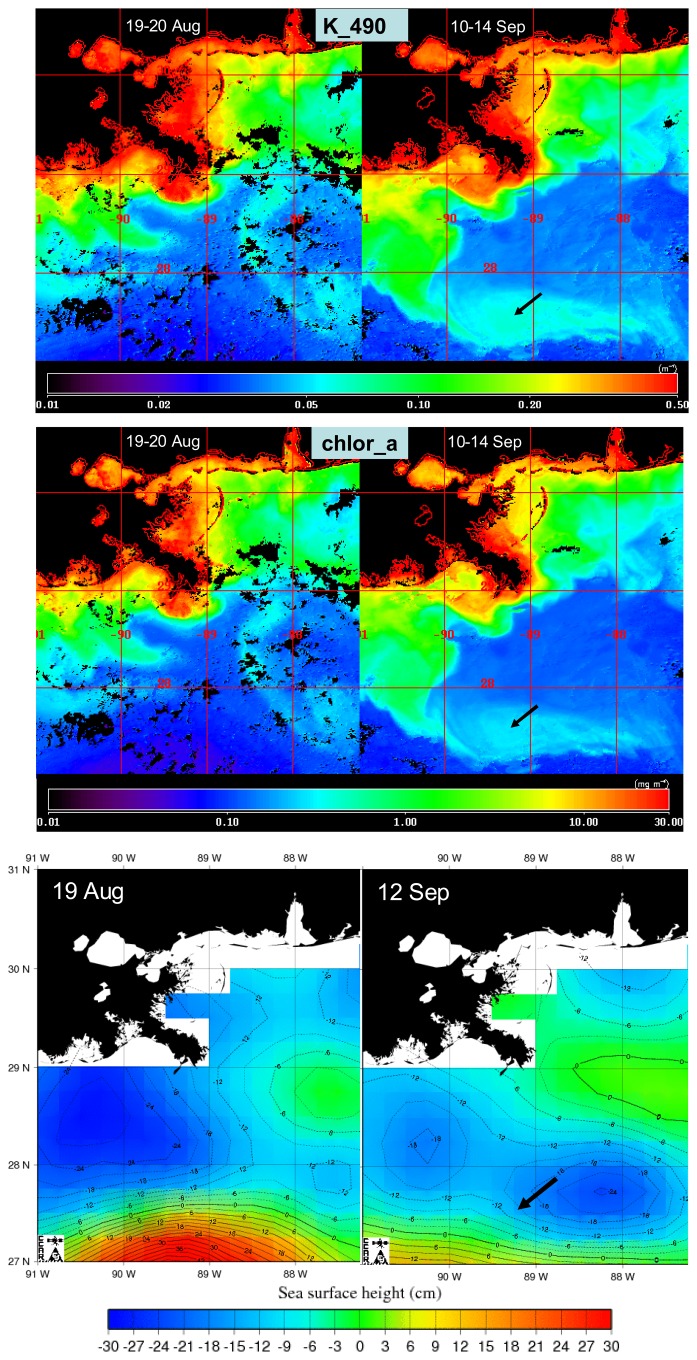
The top two panels show MODIS Aqua high resolution composite images of diffuse attenuation at 490 nm (K_490, upper panel) and chlorophyll (chlor_a, middle panel) for periods approximately two weeks prior to and two weeks after Hurricane Katrina made landfall on the northern Gulf coast. Units of K_490 were m^-1^ and units of chlor_a were mg m^-3^. The lower panel shows the mesoscale sea surface height anomaly (SSHA) in cm for similar periods. The black arrows provide a spatial reference.

A comparison of MODIS Aqua true color images two days prior (27 August) and two days (31 August) after Hurricane Katrina made landfall on the Gulf coast revealed substantial increases in turbidity especially in the inner shelf region offshore of the Mississippi Gulf coast (Figure 2). The imagery reveals a large area over the inner shelf of brown and presumably sediment-laden water extending well east of Mobile Bay. In a true color image on 3 September, five days after the storm had made landfall, the brown coloration had largely dissipated (Figure 3). However, there were still large areas of discolored water extending over much of the shelf off the Mississippi and Alabama coast (Figure 3, top panel). The distribution of this was also evident in the satellite-derived diffuse attenuation coefficient at 490 nm (K_490, Figure 3, lower panel). Elevated levels of K_490 of 0.1-0.2 m^-1^ were associated with what appeared to be two major extrusions extending out from the inner shelf. The effects of the storm on water column turbidity in shelf waters were not persistent as shown by a comparison of composite images for K_490 and satellite-derived chlorophyll a (chlor_a) for 19-20 August and 10-14 September (Figure 4, upper panel). Optical conditions in the inner shelf, as represented by values of both K_490 and chlor_a were not noticeably different two weeks prior to and two weeks after the storm (Figure 4), an indication that, based on satellite viewable properties.

An increase in K_490 and chlor_a was evident in offshore waters south of 28°N, consistent with offshore entrainment of coastal water or increased phytoplankton biomass (Figure 4, upper and middle panels). The feature extended offshore west of 90°W and extended from west to east as a filament south of 28°N. An examination of the SSHA for the same time periods revealed that the region of elevated K_490 and chlor_a in offshore waters coincided with the edge of a region of negative SSHA (Figure 4, lower panel), an indication of a cyclonic mesoscale circulation feature (cold-core eddy). This was bounded to the south by a region of relatively high SSHA characteristic of anticyclonic circulation related to the Loop Current (LC).

**Figure 5.**
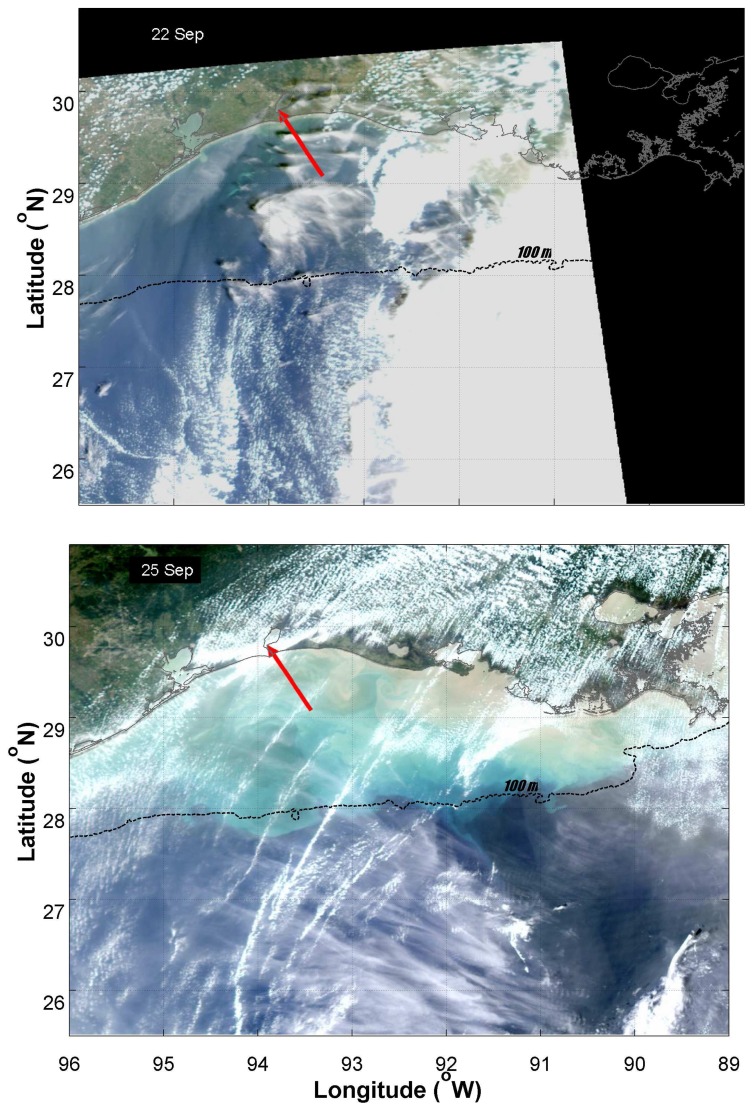
MODIS Aqua high resolution true color images of the northern Gulf coast region approximately two days prior to (top panel) and one day after (bottom panel) Hurricane Rita made landfall. The red arrow indicates the approximate location of the point of landfall. The dashed line corresponds to the 100 m isobath, which is representative of the shelf edge.

An examination of true color imagery prior to and after Hurricane Rita made landfall revealed a similar pattern of intense resuspension over Louisiana and Texas shelf waters two days after the storm made landfall (Figure 5). Large areas of brown water, presumably carrying resuspended sediment and dissolved materials, were particularly evident to the east of the point of landfall. This region corresponded to the outflow region of the Atchafalaya River, which may have contributed to the observed turbidity. More brightly colored waters exhibiting enhanced reflectance extended out to the shelf edge (Figure 5).

**Figure 6.**
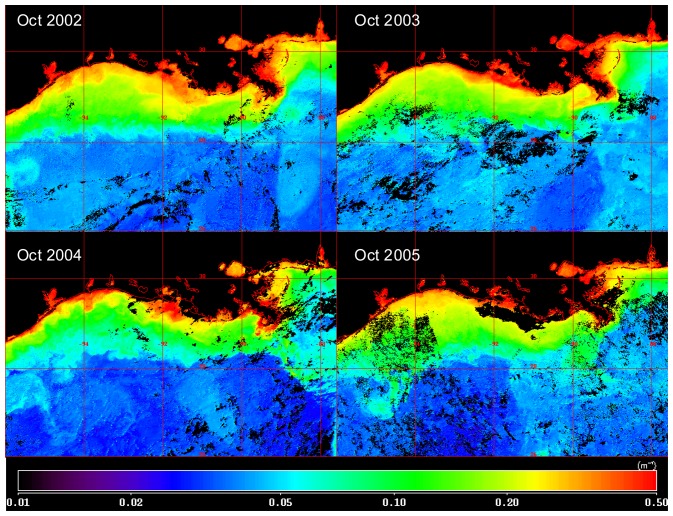
MODIS Aqua 1km resolution composite images of diffuse attenuation at 490 nm (K_490) in the northern Gulf coast region during fall 2002 – 2005. Dates included in composites imagery were 27 Sep – 1 Oct for 2002, 2-6 October for 2003, 30 September – 4 October for 2004, and 2-6 October for 2005. In the case of 2002 earlier dates were used to avoid the impacts of Hurricane Lili, which made landfall on 3 October 2002. Black pixels over water correspond to areas masked due either to cloud cover or invalid radiance retrievals.

**Figure 7.**
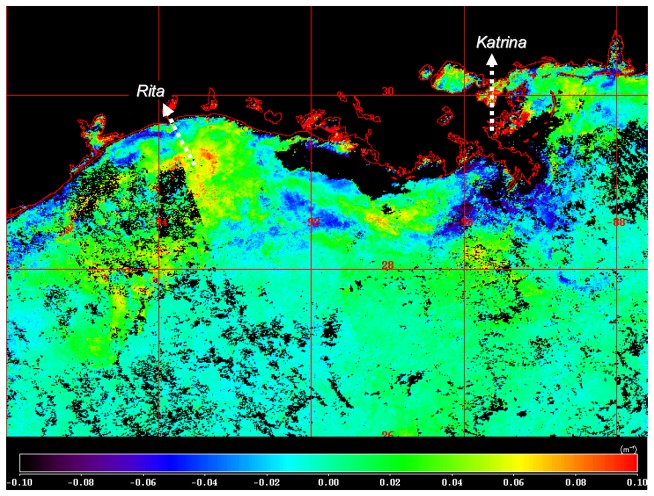
Image showing difference between the October 2005 composite image in Figure 6 and interannual mean for images from October 2002-2004. Values greater than zero correspond to pixels with higher values in 2005 than the 2002-2004 interannual mean. White arrows show approximate path of storms as they made landfall.

**Figure 8.**
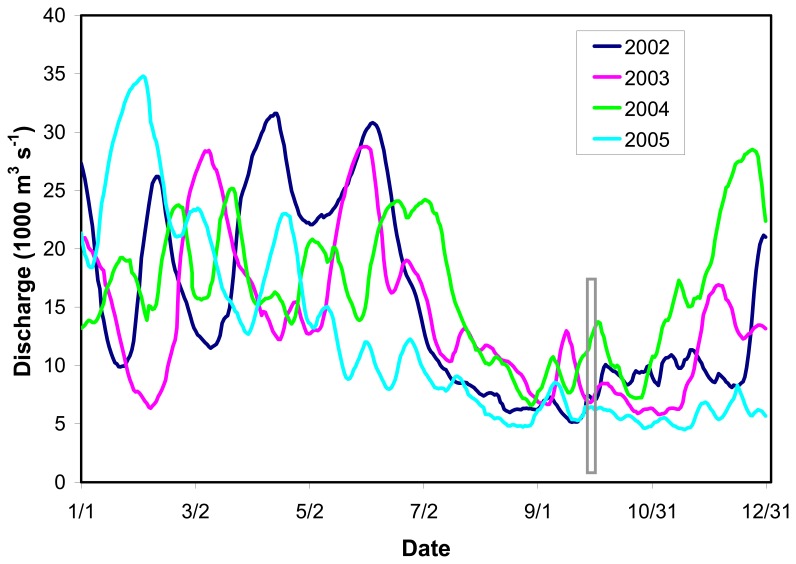
Mississippi River discharge at determined at Tarbert Landing, MS. Data were provided courtesy of the U.S. Army Corps of Engineers. The gray rectangle denotes the early October period corresponding to the imagery in Figures 6 and 7.

To quantitatively evaluate the extent to which the storms altered water column properties in 2005 relative to previous years, we compared composite images of K_490 during the fall, generally around the first week of October for the years 2002 – 2005 (Figure 6). For 2002, earlier dates were used corresponding to the last week of September to avoid the effects of Hurricane Lili, which made landfall on the Louisiana coast on 3 October 2002. Spatial patterns in K_490 were similar for all years, showing a strong cross-shelf gradient and localized areas of high turbidity in the vicinity of the Mississippi and Atchafalaya rivers (Figure 6). An image showing the difference between values of K_490 for October 2005 compared to the mean for 2002-2004 revealed higher levels over the shelf off Louisiana and Texas (Figure 7). The area of elevated K_490 corresponded to the vicinity of landfall for Hurricane Rita. In contrast, the area around the Mississippi River delta close to where Katrina made landfall exhibited lower values of K_490 in 2005 compared to the previous three year mean. An examination of Mississippi River discharge for the 2002-2005 period (Figure 8) revealed that discharge in late 2005 was lower than previous years and this may have contributed to the lower turbidity in the vicinity of the delta.

**Figure 9.**
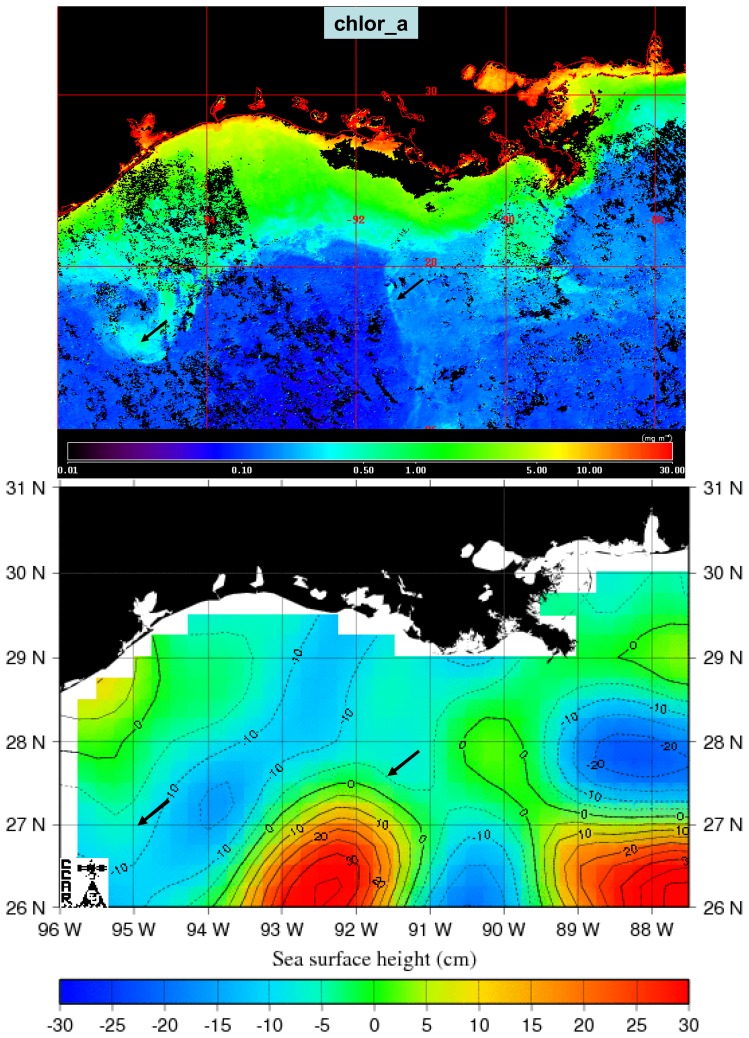
Upper panel: composite image of chlor_a derived from MODIS Aqua 1 km resolution imagery for the period 2-6 October 2005. Lower panel: historical mesoscale SSHA for 4 October 2005. Black arrows provide spatial reference to features.

The higher values of K_490 observed off Texas and Louisiana in 2005 coincided with regions of relatively high chlor_a (Figure 9, upper panel). Offshore patterns in chlor_a features were related to patterns observed in SSHA (Figure 9, lower panel). A region of high chlor_a extending off the Texas shelf was associated with the boundary between regions of high and low SSHA (black arrow in the western portion of image in Figure 9). An area of high SSHA, indicative of an anticyclonic or warm-core eddy, was associated with low values of chlor_a (black arrow near center of image in Figure 9).

## 4. Discussion

Satellite imagery revealed dramatic changes in water column properties over the shelf following hurricanes Katrina and Rita, as evidenced by substantial increases in turbidity seen in true color imagery. Subsequently, we observed a relatively rapid return of shelf water quality to pre-storm conditions. Rapid recovery of water column conditions following hurricane events has been reported in other previous investigations. For example, moored time series observations of beam attenuation on the New England shelf during hurricanes Edouard and Hortense similarly revealed large resuspension events from a depth of ∼70 m (Chang et al., 2001), followed by a return to near pre-storm levels in 7-10 d. The observations of Hu et al. (2007) of rapid return of sea surface reflectance to pre-storm levels after about 10 d following Hurricane Dennis were also consistent with our observations. Prior studies have similarly demonstrated large disturbances in coastal sediments due to major tropical storms consistent with our observations. Allison et al. (2005) reported evidence for both erosion and deposition in sediments of the inner shelf off the Atchafalaya River following Hurricanes Lili. These authors also observed macrofaunal burrows in storm deposits suggesting rapid recolonization by benthic organisms. Goni et al. (2006) similarly observed evidence for major sediment disturbance following Hurricane Lili and noted that the magnitude of sediment and organic matter deposition following the storm exceeded annual inputs of these materials from the Atchafalaya River and coastal primary production. Consistent with our observations, Goni et al. (2006) reported that water column properties one week after the storm showed little evidence of the storm impacts aside from lower than expected inshore salinities.

An increase in chlor_a in shelf waters following Hurricane Katrina was not apparent in our observations, but increased levels were observed in offshore waters. The lack of increased chlor_a in shelf waters is at odds with prior studies that have reported increases in satellite observations of chlorophyll in shelf waters following the passage of hurricanes in the northern Gulf of Mexico (Walker et al., 2005), on the northeastern U.S. continental shelf (Davis and Yan, 2004), and in Chesapeake Bay (Miller et al., 2006). An increase in SSHA, consistent with anticyclonic circulation, occurred off the shelf after the storm and we postulate that circulation patterns limited the entrainment of offshore nutrients onto the shelf. We did observe increases in chlor_a in offshore waters that coincided with areas of negative SSHA, which are characteristic of cyclonic circulation (cold-core eddy) features. Walker et al. (2005) similarly observed enhancement of chlorophyll a concentrations in such regions following Hurricane Ivan and noted that cyclonic eddies are characterized by upward doming of isopycnal surfaces. Storm-induced vertical mixing could thus more readily entrain nutrients from depth in these features. Yuan et al. (2004) reported storm-induced injections of Mississippi River water into offshore Gulf waters that was accompanied by blooms of phytoplankton. Davis and Yan (2004) found the appearance of filaments of elevated chlorophyll following hurricane events off the northeastern U.S. to be a consistent phenomenon.

It should be noted that satellite-derived estimates of chlorophyll in coastal waters may exhibit bias in relation to the actual chlorophyll concentrations. Although the algorithms for diffuse attenuation at 490 nm (K_490, Equation 1) and for chlorophyll (chlor_a, Equation 2) have a different form, they nonetheless utilize common wavelength bands in turbid coastal waters (i.e., $R_{551}^{488}$ for chlor_a and [*L_w_*(488)/*L_w_*(551)] for K_490). Constituents that tend to increase values of K_490 such as colored dissolved organic matter and suspended sediment may interfere with the estimate of chlor_a (Darecki and Stramski, 2004). Satellite estimates of chlorophyll have, nonetheless, been shown to correctly represent spatial and temporal trends in coastal waters despite bias in absolute accuracy (Harding et al., 2005).

Our observations revealed elevated levels of K_490 in shelf waters off the Texas and Louisiana coasts following Hurricane Rita relative to prior years' conditions even after a period of 7-10 d. This may be attributable to the fact that this region receives large amounts of relatively fine grain sediments that remain in suspension for a longer period of time, particularly after an intense wind event. In support of this argument, both Allison et al. (2005) and Goni et al. (2006) reported the deposition of fine grain layer of sediments following Hurricane Lili. Another explanation for elevated values of K_490 following Rita was input of dissolved materials introduced through a combination of bottom resuspension and offshore transport of terrestrial inputs. Stimulation of growth of phytoplankton could also have contributed to elevated levels of K_490. This latter possibility is supported by observations of elevated levels of chlor_a in this same region (Figure 9). As was observed for offshore conditions following Katrina, the areas of enhanced chlor_a corresponded to regions of negative SSHA, presumably associated with cyclonic circulation and conditions that would favor upwelling and enhanced nutrient entrainment. Stimulation of phytoplankton growth in offshore waters has been reported by other investigators. As noted previously, Walker et al. (2005) observed increases in chlorophyll in offshore waters in the Gulf of Mexico following Hurricane Ivan in association with cyclonic eddies. Gierach and Subrahmanyam (2008) noted offshore enhancement of chlorophyll in Gulf of Mexico waters that were associated with cyclonic eddies following hurricanes Katrina, Rita and Wilma. They attributed the elevated chlorophyll to both enhanced growth due to nutrient entrainment into surface waters as well as entrainment of phytoplankton from the deep chlorophyll maximum. Enhancement of chlorophyll in the wakes of hurricanes has also been observed in the Sargasso Sea region (Babin et al., 2004).

Lower levels of K_490 in October 2005 were observed relative to the previous three year mean off the Mississippi River delta and this could be at least partially explained by lower river discharge during the preceding months. Indeed, work by Salisbury et al. (2004) reported a strong correlation between surface particle concentrations derived from Sea-viewing Wide Field of View Sensor (SeaWiFS) ocean color imagery and river discharge in the vicinity of the Mississippi River delta. Contrastingly, they found that surface particle concentrations over the Louisiana shelf were more strongly correlated with wind stress, and attributed this to the relatively shallow and wide bathymetry of the Louisiana shelf. Such findings are consistent with our observations of extensive sediment resuspension over large areas of the shelf off Texas and Louisiana following Hurricane Rita.

## 4. Conclusions

Our results illustrate extremely high turbidity levels occurring immediately following the passage of intense tropical storms over coastal waters and were indicative of major resuspension events and, in some cases, an apparent offshore extrusion of material. Independent observations from other studies provide evidence of a high level of sediment disturbance and redistribution consistent with our observations. Our findings suggest that shelf water column conditions, at least those visible with ocean color imagery, were restored relatively rapidly to pre-storm levels off the Mississippi Gulf coast. However, elevated levels of K_490 and chlor_a were observed in offshore waters and were associated with cyclonic circulation features. Levels of K_490 during early October 2005 were higher in relation to the prior three year mean in regions off Texas and Louisiana in the vicinity of Hurricane Rita's path. In contrast, values were lower off the Mississippi River delta, and this was attributed to unusually low river discharge during fall 2005. The elevated levels off Texas and Louisiana were speculated to be the result of persistent fine grain sediment or dissolved materials that remained in the water column following the storm; higher diffuse attenuation may also have been associated with enhanced phytoplankton biomass associated with cyclonic circulation features and associated vertical mixing as well as the possible offshore delivery of shelf water and associated nutrients.
